# Use of Cohesive Approaches for Modelling Critical States in Fibre-Reinforced Structural Materials

**DOI:** 10.3390/ma17133177

**Published:** 2024-06-28

**Authors:** Vladislav Kozák, Jiří Vala

**Affiliations:** Institute of Mathematics and Descriptive Geometry, Faculty of Civil Engineering, Brno University of Technology, 602 00 Brno, Czech Republic; vladislav.kozak@vut.cz

**Keywords:** extended finite element method, fibre composites, nonlocal approaches, crack resistance, 62.20.mt, 46.50.+a

## Abstract

During the operation of structures, stress and deformation fields occur inside the materials used, which often ends in fatal damage of the entire structure. Therefore, the modelling of this damage, including the possible formation and growth of cracks, is at the forefront of numerical and applied mathematics. The finite element method (FEM) and its modification will allow us to predict the behaviour of these structural materials. Furthermore, some practical applications based on cohesive approach are tested. The main effort is devoted to composites with fibres and searching for procedures for their accurate modelling, mainly in the area where damage can be expected to occur. The use of the cohesive approach of elements that represent the physical nature of energy release in front of the crack front has proven to be promising not only in the direct use of cohesive elements, but also in combination with modified methods of standard finite elements.

## 1. Introduction

The question of ensuring the safety of structural elements regardless of their size and predicting their service life is increasingly being asked in connection with the development of advanced materials, devices, structures and their components not only in the field of engineering or construction. This can depend on the occurrence of defects that can arise during the production phase or during their operation and loading under external conditions. The field of science, summarised as fracture mechanics, combining continuum mechanics with materials engineering, tries to predict the formation and further development of defects in structures. It is a complex defect–stress–material relationship, not included in both classical and advanced approaches to the modelling of composite structures by [1]. To understand relationships and extend lifespan, it is not only necessary to modernise procedures or relevant standards, but also to use new ones with more accurate numerical methods, which with increasing probability can predict the behaviour of materials even under complex operating conditions. The behaviour of bodies containing defects has been described and predicted using fracture mechanics.

There are two basic approaches to derive the conditions at the moment of initiation of unstable crack propagation. The first one uses the weakest link theory, following [2], and the second one considers the accumulation of damage during the life cycle, as discussed by [3]. Failure of structural materials is a continuous process in which the phases of plastic deformation, nucleation and initiation of cracks are intertwined. The final stage of failure development of bodies, which is the subject of fracture mechanics investigation, is crack propagation (unstable or stable). Attention is paid to understanding this behaviour of structural materials to the deterministic approaches and to the computational modelling of such processes in the case of several representative types of composites, unlike various types of probabilistic considerations by [4,5,6,7], or discrete element techniques by [8,9], or even those relying on techniques of artificial intelligence as [10]. This article uses the long-time experience of authors in this research field, confronted with new models, methods and results developed in recent years.

As a result of the fracture, the structure breaks into two or more parts. We define a crack as a violation of the cohesion of bodies along a surface bounded by a curve, which is either closed or ends on the surface of the body. An important problem is to define criteria that will unambiguously determine the state of existing microcracks, for example, those created during manufacturing processes, before these propagate further. The concept of the stability criterion can be extended to the case of general stress concentrators, in which case, the conditions under which the crack starts to propagate from the considered concentrator has to be selected. In general, it is possible to formulate stability criteria and propagation criteria on the basis of energy by [11,12,13], or using the strain energy density, crack driving force, *G*, *J* integral, or based on the stress and strain at the crack tip (stress intensity factor concept, crack opening). For linear fracture mechanics, the more frequently used stability criterion is the $K_{IC}$ criterion (the $G_{IC}$ criterion can be used equivalently). For nonlinear fracture mechanics, the $J_{IC}$ criterion by [14] was formulated and with connection to cohesive energy by [15], or the crack tip opening displacement (CTOD) condition, with a series of criteria for combined stress, suggested by [16,17,18]. Connection of the $J_{IC}$ integral with a special cohesive element for dynamic crack propagation can be seen in [19].

From a mathematical point of view, the complete system of partial differential equations of evolution, both in its classical differential form and in its variational or weak integral form, relies on the conservation principles of classical thermomechanics, supplied by appropriate constitutive equations, as presented by [20]. Since such formulations work with function spaces of infinite dimension; thus, most computational approaches to real engineering problems need some discretisations both in the Euclidean space, 3-dimensional in general, and on certain time variables, even for a seemingly static, simplified evaluation of fracture development. The conservation principles contain both the total strain tensor ε and the stress tensor σ, both represented by symmetric square matrices of order 3. Since the values of components $\sigma_{ij}$ of σ with i,j∈{1,2,3} depend on the choice of a Cartesian coordinate system, for the following considerations, it is useful to introduce also three invariants of σ (independent of the choice of Cartesian coordinates) $\sigma_{I}$, $\sigma_{II}$ and $\sigma_{III}$; the first (linear) one is $\sigma_{I}=\sigma_{11}+\sigma_{22}+\sigma_{33}$; for the derivation of their complete set, see [21]. Most computational tools ϵ are decomposed into four components, denoted as $\epsilon^{e}$, $\epsilon^{p}$, $\epsilon^{c}$ and $\epsilon^{\theta }$, referring to elastic, plastic, creep and thermal ones; moreover, an appropriate incorporation of the damage process is expected.

In particular, in the simplified small deformation theory for isotropic materials, using the standard Kronecker symbol $\delta_{ij}=1-\mathrm{sgn}\;\left|i-j\right|$, for purely elastic deformation, the following equation

(1) $$\varepsilon_{ij}=\varepsilon_{ij}^{e}=\frac{1+\nu }{E}\;\sigma_{ij}-\frac{\nu }{E}\;\sigma_{I}\delta_{ij}$$

can be written for all i,j∈{1,2,3}; this relation is the famous empiric Hooke’s law, containing a couple of material parameters (E,ν): *E* is the Young’s modulus and ν is the Poisson’s ratio. To derive ε for $\varepsilon \neq \varepsilon^{e}$, unlike (1), some appropriate decomposition of ε must be suggested: in most computational tools, ε is considered as a simple sum of $\varepsilon^{e}$, $\varepsilon^{p}$, $\varepsilon^{c}$ and $\varepsilon^{\theta }$. Nevertheless, proper nonlinear formulations, namely, for the elastoplasticity theory, see [21], and in the more general context for the theory of structured deformation by [22], revisiting the original idea in [23], require more complicated multiplicative decompositions, with non-classical material characteristics and internal variables.

The irreversible component $\varepsilon^{p}$ is activated (takes non-zero values) after a certain function $F(\sigma_{1},\sigma_{2},\sigma_{3})$ for principal stresses reaches some prescribed value $\sigma_{*}$: e.g., for metals, the von Mises criterion with

(2) $$F\left(\sigma_{1},\sigma_{2},\sigma_{3}\right)=\sqrt{\frac{1}{2}\left({\left(\sigma_{1}-\sigma_{2}\right)}^{2}+{\left(\sigma_{2}-\sigma_{3}\right)}^{2}+{\left(\sigma_{3}-\sigma_{1}\right)}^{2}\right)}$$

is used frequently, with $\sigma_{*}$ considered as the yield strength of material in simple tension; the left-hand side of (2) can be then understood as an equivalent stress. Then $\varepsilon^{p}$ can be evaluated from the associated plastic flow rule, following [21,24]; all additional material parameters are expected to be identified from well-prepared experiments. The superposition of $\varepsilon^{c}$ and $\varepsilon^{e}$ relies on the Kelvin–Voigt model typically; $\varepsilon^{c}$ is evaluated as a function (in general nonlinear, especially in the well-known Norton power-law model of creep) of stress components or invariants, frequently from the deviatoric part $\sigma^{\prime }$ of σ, i.e.,

(3) $$\sigma_{ij}^{\prime }=\sigma_{ij}-\frac{\sigma_{I}}{3}\delta_{ij}\;,$$

with i,j∈{1,2,3} again, and its Euclidean norm *S* satisfying

(4) $$S^{2}=\sum_{i,j=1}^{3}\sigma_{ij}^{\prime }\sigma_{ij}^{\prime }\;.$$

Most simplified evaluation of $\epsilon^{\theta }$ can rely on the linear dependence of $\epsilon^{\theta }$ on the prescribed temperature change, using certain thermal expansion coefficient(s); deeper analysis results in multi-physical models including both mechanical deformation and thermal transfer, which exceeds the scope of this article.

In the field of both linear and nonlinear fracture mechanics, special curvilinear integrals are introduced as its substantial ingredients, namely, for the analysis of crack tip phenomena. The above-mentioned energetic contour path integral, called *J*-integral, comes from [14,25]. An alternative way, utilised by [26,27,28,29], derives the crack propagation from the *C*-integral, working with energy rate. For a better understanding of such individual types of curvilinear integrals, the theoretical Warp3D software system manual [30] can be recommended. However, in the case of multiaxial stresses, the one-parameter fracture mechanics, referring to the analysis of the stress around the crack front, may be insufficient; the multi-parameter fracture mechanics, followed by [31,32], work with such concepts as *T*-stresses or *Q*-parameters.

In the history of FEM-based numerical simulations, some simple approaches to modelling of crack propagation should be mentioned, namely, the element deletion approach by [33] and the node release method by [34], upgraded as the continuous remeshing technique of [35]. Another model of the accumulation of damage is processed by [36]. As an example of using damage mechanics for ductile failure, the Gurson–Tvergaard–Needleman (GTN) model can be pointed out: see [37,38]. Based on the evaluation of the performed experiments, it is recommended to introduce two (or three) optional parameters $q_{1},q_{2},q_{3}$, usually $q_{1}=1.5$, $q_{2}=1$, $q_{3}=q_{1}^{2}$. A careful analysis of the influence of these parameters on the development of damage was carried out by [39]. Here, $\sigma_{YS}$ is the yield stress of the matrix material, $\sigma_{m}$ is the principal (hydrostatic) stress and F is the volume fraction of voids. The energy balance of such so-called complete model by [40], implemented as a user procedure in Abaqus software, as documented by [41], gives an equation for plastic potential describing plastic flow in porous materials

(5) $$\frac{2S^{2}}{3\sigma_{YS}^{2}}+2q_{1}F\cosh\frac{3q_{2}^{2}\sigma_{m}}{2\sigma_{YS}}-q_{3}F^{2}=1\;,$$

cf. (3) and (4). For practical calculations, the volume fraction of voids F in (5) is usually replaced by the effective volume fraction $F^{*}$, introduced as

(6) $$F^{*}=F_{c}+\frac{F_{u}^{*}-F_{c}}{F_{F}-F_{c}}\left(F-F_{c}\right)\;;$$

here, $F_{c}$ is the critical volume for coalesce of voids, $F_{F}$ is the volume of voids at ultimate damage and $F_{u}^{*}=1/q_{1}$, as needed in (5). The effective value $F^{*}$ by (6) applies to (5) when $F_{c}$ is less than F.

FEM is widely used in the numerical analysis of initial and boundary value problems for differential equations; however, as already indicated in the article, the FEM mesh may not always be ideal for modelling crack propagation. And this is one of the most important interests in the problems of mechanics of solid bodies. The first models were established on a weak (deformation) discontinuity that could pass through the finite element mesh, using the variational principle by [42]. Other authors and researchers considered it a strong (displacement) discontinuity by modification of the principle of virtual work (which also applies to models with the traction–separation law), as [43,44], including the stability and convergence of such problems, as [45], and improving the accuracy of such modelling, namely, [46,47].

In the case of a strong discontinuity, the displacement consists of regular and amplified components, where the improved components are given a jump over a discontinuous surface by [44]. There is a modification of the basic equation, well known from the standard finite element method (FEM), for the relationship between displacements $u^{\epsilon }\left(x\right)$ on particular points *x* of the *e*-th element (three-dimensional vectors of functions in general) and displacements at selected element nodes $u^{i}$, utilising some standard shape functions $N_{i}\left(x\right)$, i.e.,

(7) $$u^{\epsilon }\left(x\right)=\sum_{i\in E_{A}}N_{i}\left(x\right)u^{i}\;;$$

here, $E_{A}$ is the set of nodes corresponding to the standard *e*-th element.

If a modification of FEM such as XFEM (extended finite element method) is considered, some more terms must be added to slightly modified displacements inside the element, and displacements at element nodes. It is the second term that points to the technique of penetration into the element and the crack movement, while the third term is failure (decohesion) and the possible direction of movement according to the preferred criteria. Therefore, (7) for the case of crack growth for two-dimensional modelling (not limited to one element) is modified into the form

(8) $$u^{\epsilon }\left(x\right)=\sum_{i\in E_{A}}N_{i}\left(x\right)u^{i}+\sum_{j\in E_{B}}N_{j}\left(x\right)H_{j}\left(x\right)+\sum_{k\in E_{C}}N_{k}\left(x\right)\sum_{m=1}^{M}\phi_{k}^{m}\left(x\right)c_{k}^{m}\;,$$

including certain specialised shape functions $N_{j}\left(x\right)$ and $N_{k}\left(x\right)$ for the intrinsic version of XFEM, unlike the extrinsic version that adopts the original functions $N_{i}\left(x\right)$ from (7). H(x) here is a certain discontinuous function with values 0 and 1, interpretable as the Heaviside function in the local crack coordinate system. Whereas the first additive right-hand-side term of (8) is identical with that of (7), the second one supports the evaluation of crack formation, working with some set of nodes $E_{B}$, and the third one is the inclusion of the local phenomena in front of the crack, working with a still other set of nodes $E_{C}$. The well-known setting with M=4, frequently used in the two-dimensional crack propagation modelling, relies on the choice of special functions $\phi_{k}^{m}\left(x\right)$ by [48].

**Figure 1:**
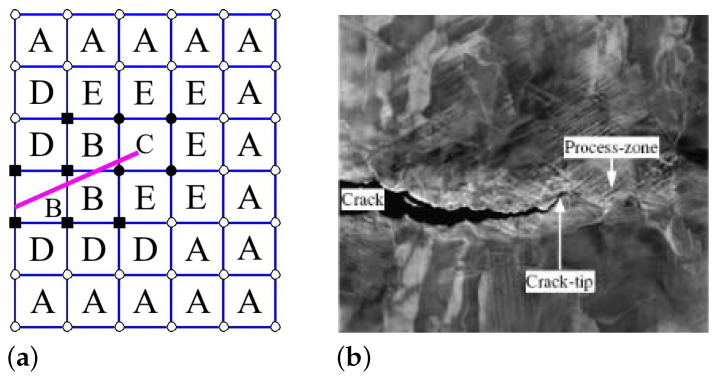
(**a**) Schematic picture describing the situation in front of the crack when using XFEM. Empty circular nodes belong to $C_{A}$, solid ones to $C_{C}$ and solid square nodes to $C_{B}$, as needed in (8), *D* and *E* are critical areas where a crack is likely to occur. (**b**) Crack in the microstructure of forged steel, the real size of the snapshot is 30 μm × 25 μm.

Figure 1a illustrates the location of areas *A*, *B* and *C*, a related model area around the moving crack; the sets of nodes $E_{A}$, $E_{B}$ and $E_{C}$ of these areas occur in (7) and (8). Figure 1b shows the real crack shape in forged steel. The progress of this approach, adopted for three-dimensional modelling at the expense of much more complicated functions $\phi_{k}^{m}\left(x\right)$, in the last years can be seen from [49].

In general, the method of discretisation in time and the extended finite element method (XFEM) can be used for the adaptive change and/or enrichment of the set of basis functions near singularities. This computational approach (involving its numerous variants with its own names and designations) already has quite a rich history with remarkable progress in recent years, as documented by [50,51,52,53,54,55], including the detailed analysis of convergence properties for various enrichment strategies by [56]. Namely, an improvement of XFEM, utilising an upgrade of (7), presented as IXFEM, is introduced by [57] to reach inexpensive computations with good geometrical accuracy and surface mesh resolution independence. Another approach relies on the simplification of “smeared” damage by [58,59,60,61,62]: instead of the detailed analysis of the initiation and propagation of particular cracks, certain damage factor, or more such factors, are analysed to evaluate the partial loss of material stiffness. Both approaches can be coupled, even for finite deformations and dynamic fracture, relying on certain nonlocal regularisation of material properties and on advanced crack tracking procedures, cf. [63,64]. Examples and classic procedures focused on modelling the growth, namely, the formation of crack propagation, can be found in [65]. The purpose of this paper is to show the results of a study focused on the occurrence of damage heterogeneous materials. Attention is paid to the direct application of the finite element method to different types of materials in order to find critical parameters determining behaviour of materials at the damage process.

## 2. Materials and Methods

Three groups of materials to which the cohesion approach can be applied were selected for the following modelling:

- Forged steel;
- Ceramics with fibres;
- Cementitious composites.

### 2.1. Forged Steel

**Figure 2:**
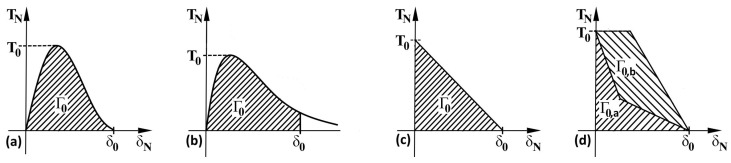
Traction–separation law for (**a**) plastic behaviour of materials, (**b**) modified plastic behaviour of materials, (**c**) elastic bilinear response, (**d**) composites with fibres. $T_{N}$ here is the peak of (normal) stress, $\delta_{N}$ is the displacement in the direction of the crack growth, $\Gamma_{0}$ is the energy which can be represented by the fracture toughness, $\Gamma_{0a}$ and $\Gamma_{0b}$ are the mean energies for composites reinforced by fibres. If total energy equals $\Gamma_{a}+\Gamma_{b}$, reinforcement has zero influence.

**Figure 3:**
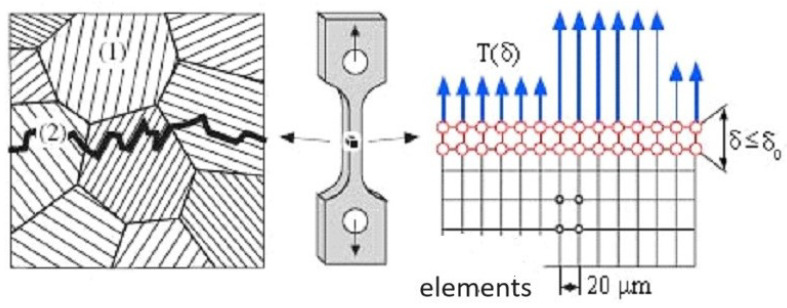
The concept of cohesion approach.

If the possibilities of cohesive approaches for modelling the behaviour of materials had been used, it would have been necessary to have the following input data available based on experiments: (i) The standard true stress—true strain curve obtained by tensile tests; (ii) allowing crack growth through the selection of initialisation criteria; and (iii) traction–separation law representing micromechanic processes. However, it can also be used to analyse certain wider aspects of material or structural behaviour, which can be verified using experiments. The individual parts of the curve show the switching on of individual mechanisms that are dominant at a given moment in the response of materials to external conditions. In a numerical model, the cohesive elements are surrounded by the classical elements. When the cohesive elements are damaged, the crack extends over the boundaries of the classical elements. Thus, a potential crack can only propagate where cohesive elements have been used and nowhere else. The shape of the traction–separation law is defined according to many authors for various types of materials and damage as can be seen in Figure 2, general concept of cohesion approach is schematically presented in Figure 3. Figure 2b shows that the zero stress value is not used in real modelling, which improves numerical stability.

**Figure 4:**
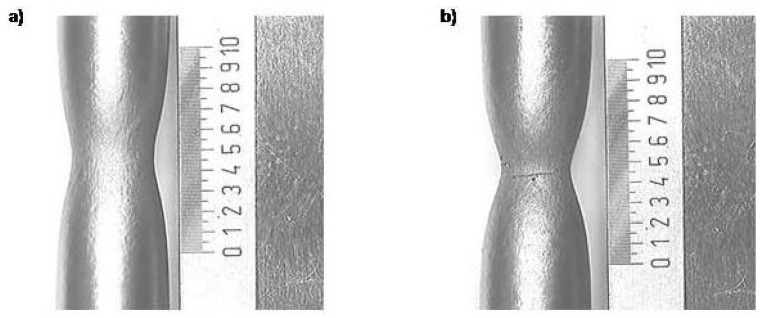
The localisation of plastic deformation (**a**) and moment of fracture (**b**) for forged steel.

**Figure 5:**
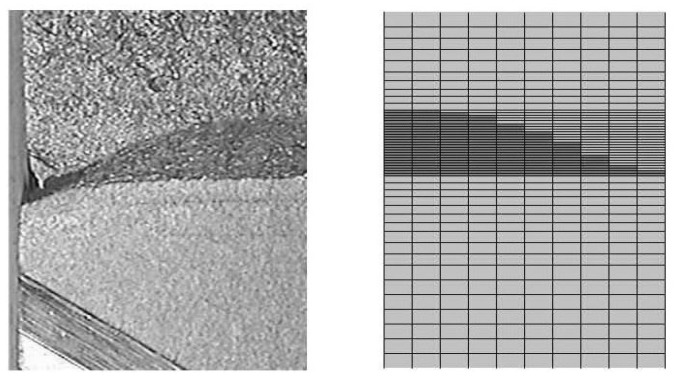
Part of the fracture surface (symmetric): experiment (**left**) and FEM using GTN model (**right**).

**Figure 6:**
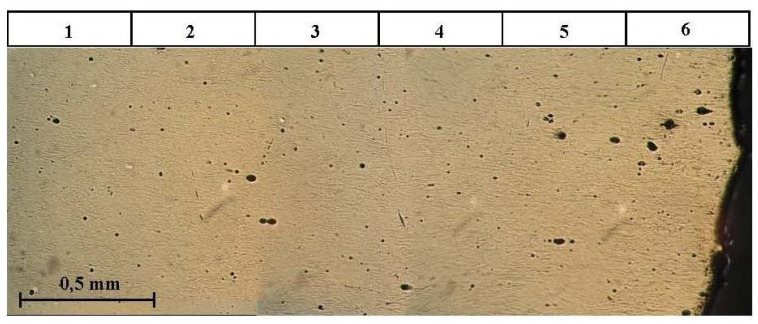
Arrangement of voids in the neck area (fracture surface is on the right), upper numbers describe the measured area.

The experimental material was supplied as block-shaped castings with dimensions of 100 × 55 × 260 mm. These blocks were annealed and then cut into blanks with dimensions of 30 × 55 × 260 mm, from which test bodies for the three-point bending test and cylindrical test bodies of the circular cross-section for the static uniaxial tensile test were made. The initial measured length $L_{0}$ = 26 mm and the diameter of the tested length *d* = 6 mm. During the static tensile test, an extensometer MTS 634-12F-51 was used; during the test, both elongation and contraction were recorded at a crossbar speed of 2 mm per minute. The chemical composition of the material in weight percent was as follows: 0.09 C, 1.18 Mn, 0.37 Si, 0.01 P, 0.12 Cr, 0.29 Cu, 0.29 Ni, 0.03 Mo and 0.028 Al. Yield strength at room temperature was 250 MPa. Prominent localisation of plastic damage for forged steel is presented in Figure 4, examples of GTN modelling can be seen in Figure 5, and Figure 6 was used for the determination of basic model parameters.

**Figure 7:**
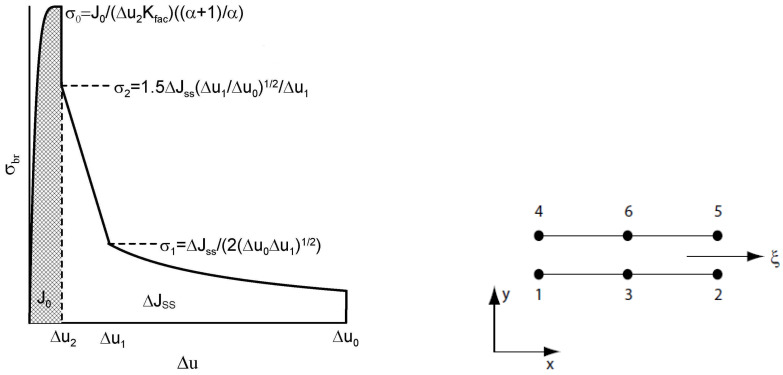
The traction–separation law for fibre composites and schematic diagram for 2D cohesive element, $J_{0}$ is the energy before the crack growth starts.

In practice, the cohesive model uses two types of finite elements. The first type describes a standard continuum; the second type specifies the connection between these areas. The decoupling of cohesion elements is determined by the displacement of standard elements, as can be seen in Figure 7. However, in the case of composite materials modelled using damage mechanics, knowledge of the decisive micro-mechanisms affecting damage is essential, which leads to realistic predictions corresponding to experimental results. The direction of crack propagation is also important. In the case of crack propagation in the direction perpendicular to the strengthening fibres, the damage is then determined by these following micromechanisms: (i) damage of matrix, (ii) delamination of an interface fibre versus matrix, (iii) fibre cracking, and (iv) fibre pull out including bridging. However, it is not easy to describe the behaviour of the fibre–matrix interface.

The 42CrMo4 steel, which was produced in the form of a forging with a cross-section of 500 × 500 mm, was chosen as an experimental material for the area of upper threshold values. The experiments were mainly focused on obtaining the fracture mechanical characteristics, especially of the surface layers of the forging. All the results of experimental work were obtained on test specimens, the longest dimension of which was always oriented parallel to the directions of the fibres in the initial semi-finished product. Due to the assumed calculations using the FEM, hardness tests were carried out with the aim of determining the gradient of properties and microstructures in the surface layers of the forging. In the case of numerical modelling, which is focused on monitoring crack growth, it is essential to describe the material as precisely as possible, especially in front of the crack tip. The observed hardness gradient in this area is not significant. For the above reasons, therefore, for the following calculations, the material was considered homogeneous and isotropic. The necessary dependences of true stress versus true strain were obtained from tensile tests. Determination of individual parameters for the GTN model, relevant verification procedures for cohesion modelling and various approaches are listed in [66,67,68,69,70].

### 2.2. Ceramics with Fibres

Modern ceramics, such as ceramics based on silicon nitride or SiC are resistant to high temperatures and have high abrasion resistance. The influence of grain bridging on the strength and fracture toughness was found, so the grains behave as short reinforcing fibres. The prediction of crack propagation through interface elements based on the fracture mechanics approach and cohesive zone model is investigated from various damage models.

A transparent matrix with carbon fibres covered with a layer of SiC was chosen for testing, having final diameter of 0.15 mm. The matrix—polycrystalline transparent magnesium aluminate “spinel” ceramics—were obtained by hot-pressing and hot isostatic pressing. The ${\mathrm{Al}}_{2}$$O_{3}$ content of the spinel is expressed as MgO*n*${\mathrm{Al}}_{2}$$O_{3}$ with n=1.5.

**Figure 8:**
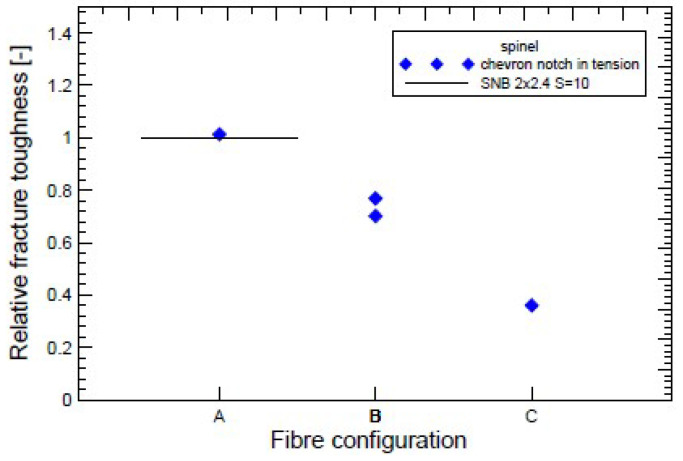
Experimentally determined fracture toughness by tension test and by the standard three-point bend test for three configurations.

**Figure 9:**
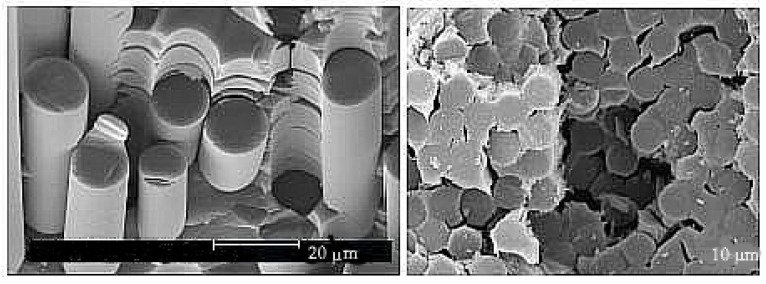
Images of the microstructure of ${\mathrm{Si}}_{3}N_{4}$.

For the following experiments, a plate with a thickness of 5 mm and a square dimension of 50 × 50 mm was made. The reinforcing fibres were placed into the middle plane of the plate periodically with nominal fibre to fibre at a distance 4 mm. The position of the fibres was very important because the experimental material was cut into rectangular shapes with respect to the own position of these fibres. Three configurations were used: samples without fibres (A), made in the same way as samples containing fibres, samples with fibres symmetrically placed in front of the tip with a sharp chevron notch (B), and finally samples with a fibres in the middle of the chevron notch (C). The properties of these microstructures are easy to spot as fracture toughness; the results are presented in Figure 8 and images of the microstructure are shown in Figure 9.

For the model case of crack propagation, the ideal direction is perpendicular to the reinforcing fibres when practically all basic damage mechanisms are activated, as are matrix cracking, delamination of interface fibres (grains) and matrix, fibre (grain) cracking, and pull out. Under extreme operating conditions, such as high temperatures and corrosive environments, where elements are subjected to high cyclic contact stresses, the modelling and life prediction are more complicated. It is well recognised that the properties of ceramics can be enhanced by suitably tailoring the microstructure based on realistic application and working conditions. Namely, the effects of tailoring the grain structure on the fracture toughness of silicon nitride were demonstrated in [71]. Similarly, the influence of boundary phase manipulation and the effect of grain bridging on the strength and toughness were illustrated in [72]. To understand crack growth mechanisms in ceramic materials, resistance curve (*R*-curve) is often used, and it helps to apply the given type of ceramics in a targeted manner in technical practice by [73]. However, more recently, it has been determined that some mechanisms cannot adequately account for the steep-rising crack growth resistance curves (*R*-curves). The development of advanced methods for determining the bridging stresses is documented in [74,75,76].

### 2.3. Cementitious Composites

Fibre-reinforced cementitious composites have appeared in the construction industry in recent years as modern materials (e.g., concrete reinforced with steel fibres), preventing tensile stress and deformations as sources of unwanted micro- and macro-cracking. The mechanical properties of such composites are determined by the choice of volume fraction of the matrix and fibres; all this is dependent to technological procedures, e.g., special compaction and eventual hardening of the matrix and some procedures (e.g., compaction) and to the early age treatment, as well as by the bond–slip interface relations, cf. [77,78,79,80,81]. The properties of the resulting structure using both destructive and non-destructive methods could be determined from the early-age matrix or obtained from the crushed part of the existing structure.

The volume fraction of particles can be evaluated accurately while obtaining information related to particle orientation is complicated. Moreover, such experiments with many constructions are not allowed by technical standards. This is obviously the impetus for using some reliable non-destructive or (at least) semi-destructive methods of measuring, for example, the orientation of fibres or determining the proportion of fibres, usable in situ, manipulation of homogeneity and isotropy and determining other parameters, and the overall structure of the material.

A crucial problem of the last few decades is the development of a computational model for the complex hydro-thermo-chemo-mechanical behaviour of cement-based composites, where the cement particles are activated by water and subsequently form the structure of the cement matrix. However, this means that subsequently, the deformation, stress and volume of the material are constantly changing, without the influence of external mechanical load. Every cement particle can be assumed to initiate hydration from the moment that it comes into contact with water. Water diffuses through the hydrate layer and reaches the surface cement particles and initiates a chemical reaction with the cement.

**Figure 10:**
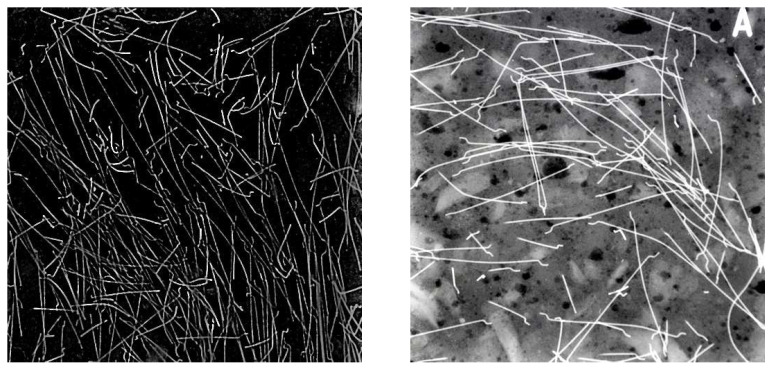
X-ray diagrams of cementitious composite with steel fibres, scale enlarged twice for rigth image.

**Figure 11:**
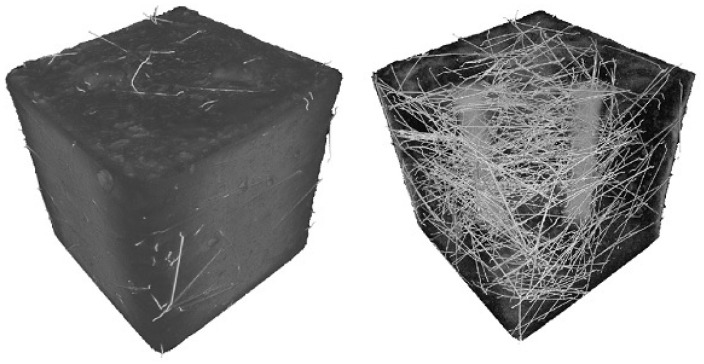
X-ray image of a concrete specimen 150 × 150 × 150 mm.

And where can most problems be found in the computational modelling of early-age behaviour of cement-based materials? Their origin is certainly in the non-periodic structure of the material, created as a result of relatively complicated chemistry response as well its different mechanisms of deformation in stress and tension, more in [82,83], including a high probability of fracture, imprecisely defined boundary conditions according to the external environment, and the necessity of coexistence with normal or pre-stressed reinforcement or other reinforcement particles, see Figure 10 and Figure 11. For more information on cementitious composites for engineering applications see the article [84]; for mechanical properties of fibre concrete, see [85]. The article focused on the preparation of composites with magnetically aligned metal fibres [86] and the authors’ practical application shows the way to the preparation of construction elements with directional properties.

### 2.4. Numerical Adjustment

As has already been mentioned several times in this text, we consider a body having crack perpendicular to the direction of oriented fibres. In the case of fibre composites, where bridging stress $\sigma_{br}$ is the dominant mechanism determining crack propagation and arrest, a relation between the bridging stress $\sigma_{br}$, see (9), and the fracture energy, then by means of fracture mechanics, can predict crack growth and propagation. The law is identical in every point of the bridging zone. Under shock loading, fibres are damaged, so the existence of a characteristic opening $\delta_{0}$ is assumed, which determines the moment when the bridging effect disappears.

To put it simply, it is energy, which has to act on the body to crack growth, which can occur at a given moment. The bridging law (e.g., in the case of materials with fibres) is accepted as another material characteristic

(9) $$\sigma_{br}=\frac{\Delta J_{ss}}{2\delta_{0}}\sqrt{\frac{\delta_{0}}{\delta }}\;.$$

Microstructural properties can be characterised using a geometric factor representative volume element (RVE) determining the range of action of the given mechanisms and thus also its representation using a cohesive element. Crack propagation through the element is controlled by the fracture mechanic and by the cohesive model. This model is simpler than the classical models and its parameters are given experimentally [87,88,89,90,91].

Several steps are necessary due to the numerical implementation: (i) remove of the stress singularity at $\delta_{u}=0$ and (ii) incorporate the initial fracture strength $J_{0}$ to improve the traction–separation law, as can be seen in Figure 7. $J_{SS}$ represents the total fracture energy without $J_{0}$. Following parameter $K_{\mathrm{fac}}$ in (10), see Figure 7, indirectly describes the peak used in traction–separation law

(10) $$K_{\mathrm{fac}}=\frac{J_{0}}{\sigma_{0}\Delta u_{2}}\left(\alpha +\frac{1}{\alpha }\right)\;,$$

where parameter α is in the range [1,100]. An element consists of two quadratic line elements for 2D planar elements or two quadratic planar elements for 3D. To indicate the determination of the new cohesive element and to explain its formation, only 2D is presented. The two surfaces of the contact element initially lie on top of each other in the initial phase and are in an unloaded deformation state. The relative displacements of the element surfaces produce normal and shear displacements, depending on the constitutive equation. Now assume a quadratic linear element for 2D simulations. This element has 12 degrees of freedom, and the nodal displacement vector is given by (11), which generates the final stiffness matrix 12 × 12 for 2D and 48 × 48 in the generalised case for 3D. A convenient design of the cohesive element is initiated by the following set of equations: (11) for nodal displacements, (12) for displacement jumps, (13) for nodal displacement jumps on adjacent layers, (14) for displacement jumps, via shape functions, (15) for corresponding shape functions, up to the desirable results (16) and (17).

In more detail, let us consider the displacements (doubled) between adjacent nodes in cohesive elements

(11) $$d_{N}=\left[D_{x}^{1},D_{y}^{1},D_{x}^{2},D_{y}^{2},\ldots .D_{x}^{6},D_{y}^{6}\right]\;.$$

3D cohesive element has 48 degrees of freedom. For element numbering, the standard conventions is used, then the opening of the connection element is determined as a difference in displacements between upper and lower nodes:

(12) $$\Delta u=u^{UPP}-u^{LOW}\;.$$

The opening between adjacent layers belonging to paired nodes is defined:

(13) $$\Delta u_{N}=\Phi \;d_{N}=\left[-I_{6x6}\;|\;I_{6x6}\right]\;d_{N}$$

where $I_{6x6}$ is the unity matrix having 6 rows and 6 columns, $u_{N}$ is a line vector with 6 columns. By default, the opening is interpolated to the integration points using shape functions. Let $N_{i}\left(\xi \right)$ be the shape function for node pair i∈{1,2,3} where ξ determines position in the local coordinate system −1<ξ<1. The relative displacement between the nodes within the elements is then given as

(14) $$\Delta u\left(\xi \right)=\left[\begin{array}{c} \Delta u_{x}\left(\xi \right)\\ \Delta u_{y}\left(\xi \right) \end{array}\right]=H\left(\xi \right)\Delta u_{N}$$

where H(ξ) is special matrix with 2 rows and 6 columns containing the quadratic shape function. For the 2D element, the shape of this matrix is as follows:

(15) $$H\left(\xi \right)=\left[\begin{array}{cccccc} N_{1}\left(\xi \right) & 0 & N_{2}\left(\xi \right) & 0 & N_{3}\left(\xi \right) & 0\\ 0 & N_{1}\left(\xi \right) & 0 & N_{2}\left(\xi \right) & 0 & N_{3}\left(\xi \right) \end{array}\right]\;.$$

We obtain

(16) $$\Delta u\left(\xi \right)=H\left(\xi \right)\Phi d_{N}=B\left(\xi \right)d_{N}$$

where B(ξ) has 2 rows and 12 columns and Δu(ξ) only 2 rows and 1 column. In the case of using large deformations, which is common when solving practical problems, local deformations in both standard directions are also calculated. Then it is necessary to use the centre nodes in both layers. The coordinates of the default configuration are given by the vector $x_{N}$ and the deformation state is predicted by the vector $d_{N}$; the coordinates of the reference surface $x_{N}^{R}$ are then calculated by linear interpolation:

(17) $$x_{N}^{R}=\frac{1}{2}\left[\;I_{6x6}\;|\;I_{6x6}\;\right]\;\left(x_{N}+d_{N}\right)\;.$$

It is widely known that the mechanical strength and crack resistance of fibre composites is high. Constructions made of these materials are then finer and the use of high-strength steels is significantly lower. Even damage resistance is often lower. Final damage is often initiated from surface or near-surface cracks, which fibres often prevent. A crucial problem is the design of such experiments, which are macroscopic in nature, but intended to estimate the microscopic behaviour of the material, e.g., random or deliberately preferred fibre directions. Some non-invasive procedures may be the way to apply for it, as are especially radiographic, tomographic and electromagnetic methods, working with a stationary magnetic field or with a harmonic electromagnetic field.

The procedure for using XFEM for time-dependent problems is detailed in [92,93,94]. This method has a number of modifications, even existing under different names; progress in recent years can be seen by comparing the work of [95] and modification via the boundary element method by [96] or more in [97]. A procedure for randomly oriented fibres is presented in [98]; an example of a real distribution can be found in the case of reinforced concrete. However, the models describing the formation of microcracks [99,100] when the stress field is modified by the nonlocal Eringen’s model [101] come out differently. The relationship between the matrix and the fibres realising the microscopic behaviour of the material can be described by a cohesive model, more in [102,103,104,105].

### 2.5. Computational Algorithms and Their Convergence Properties

In all sections above (as well as this one), our attention is paid to various kinds of geometric and material characteristics connected with the initiation and development of micro- and macrofractures. For reasonable computations, such characteristics must be inserted with some variational principles, such as those of classical thermomechanics, compatible with [20]: namely, mass, momentum and energy. From this point of view, to introduce a complete 3D model problem, let us consider a deformable body occupying a domain Ω in the 3-dimensional Euclidean space $R^{3}$, supplied with a Cartesian coordinate system $x=(x_{1},x_{2},x_{3})$, with the Lipschitz boundary ∂Ω, consisting of 2 disjoint parts Θ (supports) and Γ (with active surface loads). The displacements $u_{i}$ of $x_{i}$ for i∈{1,2,3}, i.e., of all points of Ω, are redistributed in time *t* from a finite time interval I; it is useful to relate them to the reference configuration of Ω for the starting time t=0; the upper dots will then denote partial derivatives with respect to *t*. Displacement rates $v=\dot{u}$ are needed, assuming the Cauchy initial conditions $u\left(0\right)=u_{0}$ for some $u_{0}$ from the standard Sobolev space of test functions $V=\{w\in W^{1,2}{\left(\Omega \right)}^{3}\;:\;w=\left(0,0,0\right)\;\mathrm{on}\;\Theta \}$. The fully dynamic (not only quasi-static) formulation works also with accelerations $a=\dot{v}$ with the additional Cauchy initial conditions $u\left(0\right)=v_{0}$, with $v_{0}\in V$ again. In the small deformation theory, we are allowed to introduce the symmetric strain tensor $\varepsilon_{ij}\left(w\right)=\left(\partial w_{i}/\partial x_{j}+\partial w_{j}/\partial x_{i}\right)/2$ for each i,j∈{1,2,3}. Taking some volume loads $f\in L^{2}{\left(\Omega \right)}^{3}$ and surface loads $g\in L^{2}{\left(\Gamma \right)}^{3}$ into account, variable in t∈I, the conservation of linear momentum, compatible with [106], reads

(18) (w,ρa)+[ε(w),σ(u,v)]=(w,f)+〈w,g〉

for all w∈V and t∈I where the symbols (.,.), [.,.] and 〈.,.〉 can be understood as scalar products in $L^{2}{\left(\Omega \right)}^{3}$, $L^{2}{\left(\Omega \right)}^{3\times 3}$ and $L^{2}{\left(\Gamma \right)}^{3}$, σ(u,v) being certain stress tensor. The aim is to find u∈V for any t∈I and corresponding *v*, or also *a*, respectively.

A constitutive equation is presented as the dependence of σ on *u* and/or *v* here. In linear elasticity, we can take σ=Cε(u) where *C* is a 4th order tensor of stiffness, containing (thanks to all symmetries) 21 independent material characteristics in general. In particular, for an isotropic medium, the set of these characteristics reduces to 2 Lamé factors (or to the Young’s modulus and the Poisson’s ratio). Some energy dissipation which can be implemented using structural damping in the Kelvin parallel viscous form σ=Cε(u)+αCε(v), containing a damping factor α, is here considered as a scalar for simplicity. Alternatively, mass damping takes ρa+βρv in (18) instead of ρa, working with another damping factor β. The risk of cracking in tension (unlike compression) can be handled applying certain damage factor D, compatible with [58], scalar in the simplest case, with values between 0 and 1, depending on certain invariants of σ and/or ε(u) (to guarantee the objectivity). This motivates the modification of (18) to the form

(19) (w,ρ(a+βv))+[ε(w),(1−D)C(ε(u)+αε(v))]=(w,f)+〈w,g〉.

As discussed by [106], to guarantee the existence of u,v,a in appropriate Bochner–Sobolev spaces, based on the method of discretisation in time and on the properties of several types of Rothe sequences, some regularisation for the evaluation of D is necessary, as that using the Eringen’s approach by [101]; for more details to regularisation, see [107]. More complicated approaches can be classified by their evaluation formulae of scalar or more complicated D and by their use of additional internal variables. Similar results can be derived even with (i) $\gamma \nabla (\vartheta -\vartheta^{\mathrm{ref}})$ added to ε(u) in (19), working with a thermal expansion coefficient γ, ϑ being the absolute Kelvin temperature, a priori known here, supplied by some reference value $\vartheta^{\mathrm{ref}}$, and (ii) $\gamma \nabla \dot{\vartheta }$ added to ε(v). Moreover, for modelling macroscopic cohesive interfaces, implementing XFEM (or similar) techniques in computational algorithms, in addition to the last right-hand-side additive term of (19), a contribution of a similar term introduced on internal interfaces (instead of Γ) can be considered, with the carefully defined analogue of *g* dependent on jumps of *u* and/or *v* on interfaces.

Another approach to such a coupled failure model is presented by [108]: it incorporates both smeared and discrete crack models through an energy-consistent thermodynamic framework. However, for the detailed development of damage, a less simplified model can be needed, with the evaluation of power of dissipation without non-physical ad hoc assumptions. One can expect much more complicated formulae at least for the evaluation of σ in (18), as well as still open problems in existence and convergence theory. Following [20], in accordance with [109], which can be seen as a substantial generalisation of [110], the thermodynamic formulation can be based on the evaluation of the power of dissipation, from the specific Helmholtz free energy, or from the specific Gibbs energy, respectively. This can cover, using appropriate internal variables, following [111], a rather large class on nonlinear thermodynamically admissible models. In the simplest case of [112], the dissipation potential φ can be reduced to a certain set of characteristic functions. The local well-posedness of such formulation is studied in [113], and the recent techniques for estimation of such potentials via crack tips are discussed in [114].

## 3. Results and Discussion

**Figure 12:**
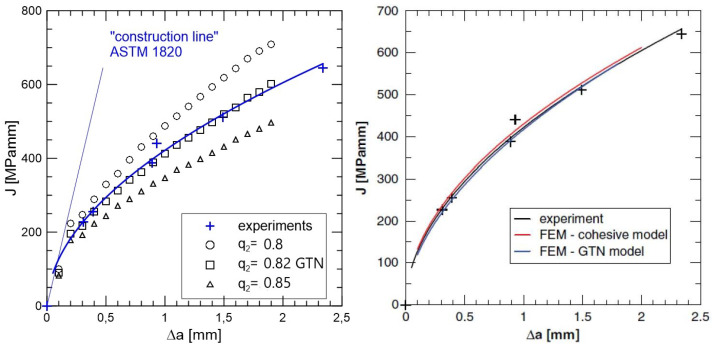
J−R curve generated by GTN model for calibrated $q_{2}$ parameter (**left**) and comparison of results obtained using both methods, i.e., GTN model and cohesion model (**right**). Non-dimensional $q_{2}$ geometric parameter from (5) is tested.

The solved problem is focused on a body with a crack under the assumption of a deformation-controlled failure mechanism. This task actually represents the use of a hybrid methodology combining numerical modelling, experiment and microscopic observation into one relatively complex whole. Prediction results for both the GTN model and the use of cohesion elements were verified using the experimentally obtained so-called J−R (*J*-integral resistance) curve, sometimes in literature known as the J−Δa, where Δa refers to crack length differences, cf. Figure 12; for the critical review of J−R curve analysis, see [115].

Briefly, the main effort is being focused on the crack propagation modelling by both methods, see Figure 12. They use the capabilities of the Abaqus and Warp3D software systems and focus more on the influence of each parameter in the models for the correct prediction of crack propagation. Like the model, the material chosen was forged steel, where both could be used due to ductile failure presented methods. The previously indicated question arises whether the procedure tested for one class of materials can be applied to another where the dimensional factor is an order of magnitude lower, e.g., composites reinforced with SiC fibres with a glass matrix. From a size point of view, the proposition of the traction–separation law requires even more understanding of the physics of ongoing events. Therefore, a user procedure was developed in the Fortran language, which has been implemented into the commercial Abaqus system.

The identification technique was chosen to study the fracture behaviour of short cracks. This procedure makes it possible to monitor almost all stages of the failure and as well in the stage of crack propagation. The detail of a very short crack (having a length of several grains) is shown in Figure 9. For real modelling, a certain calibration of the traction law is necessary using the following procedure: (a) Try first to increase $\sigma_{0}$ and decrease $\Delta u_{0}$, then minimise the bridging shape close to the maximum stress value. (b) Try to decrease $\sigma_{0}$ and increase $\Delta u_{0}$, so maximise the bridging shape close to the exponential behaviour part of this curve.

**Figure 13:**
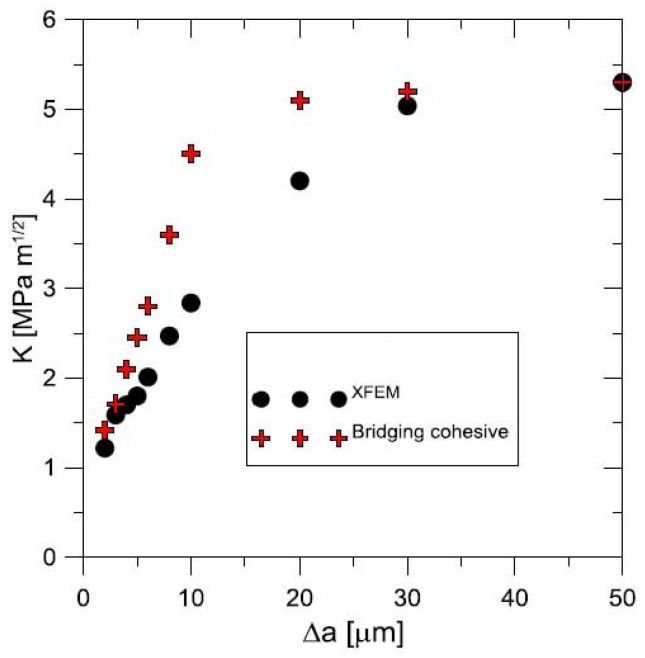
J−R curve prediction for ${\mathrm{Si}}_{3}$$N_{4}$, using created cohesive elements and standard XFEM approach including input data for tested set of silicon nitride ceramics. Due to a very small plastic zone ahead, the crack tip for cracking ceramics *K* is often used. The material parameters were $\Delta J_{ss}=64$ J/$m^{2}$, $\Delta_{c}=0.3$ μm, $\Delta_{1}$ = 0.08 μm, $dJ_{0}$ = 21 J/$m^{2}$, $\sigma_{0}=1063$ MPa.

The authors present their long-term experience with the issue and compare it with the state of knowledge in recent years. The following results deserve to be emphasised:

- Fibre bridging in crack growth realises an increase in fracture toughness associated with an increased level of strength in ceramics, similar to the interaction of grains in ductile materials.
- When modelling the behaviour of commercial ceramic as ${\mathrm{Si}}_{3}$$N_{4}$, see Figure 13, the onset and the initiation of the crack length Δa is slower when the effect of bridging into the model is introduced, which is closer to an experiment.
- It is very likely that early real bridging starts due to numerical oscillation and the obtained values of displacements after the crack initiation are smaller; see the shape of the traction–separation law in Figure 7.
- Real determination of the shape of the separation curve generates *J* – *R* prediction, at least the maximum stress $\sigma_{0}$ must be determined on the basis of careful experimental procedures.

As indicated in the introductory section, this type of modelling requires collaboration with careful experiments and techniques that see more into the microstructure. An example can be non-destructive testing of material structure via image processing (2D X-ray, 3D tomographic) and stationary magnetic and non-stationary electromagnetic approaches. For fibre cement composites, where fibres are almost randomly distributed, which is often the situation in the construction industry, there is control over the volume fraction and orientation of fibres so far only possible in the production of fresh fibre concrete mix. Metal fibres will, of course, improve the mechanical properties of concrete, especially fracture toughness, compressive strength, impact strength, and durability of structures, in addition fatigue strength, too.

A cement matrix reinforced with metal fibres was chosen for the following numerical tests. In practice, the most important case is cement composites containing short and intentionally or quasi-randomly oriented steel, sometimes ceramic or polymer fibres with primary suppression of some stress components, is introduced in [104], while a more detailed mathematical formulation is in [83]. Its numerical approach relies on a modified XFEM, where one can use as a criterion for the formation of a crack the cohesive traction–separation law. The results in the case of the implementation of the nonlocal constitutive stress–strain relation of the integral type are very interesting, see [58].

**Figure 14:**
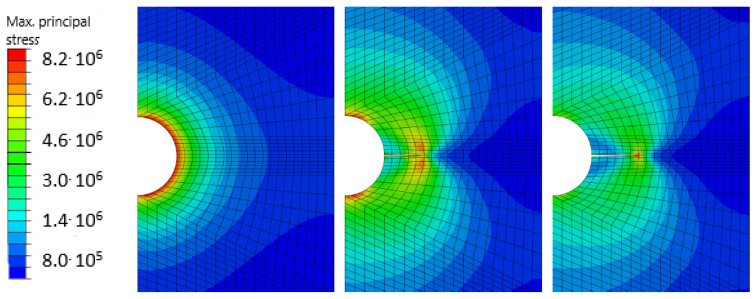
Results from the so-called Mazars’ model, following [59]. Coloured maps represent the scale from $8.2\times 10^{6}$ to $8.0\times 10^{5}$ MPa.

**Figure 15:**
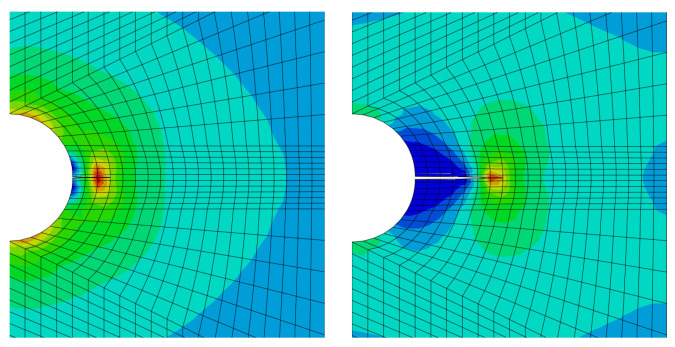
The application of the crack homogenisation with Eringen’s model, cf. [101]. Coloured maps represent the same scale as previous Figure 14.

Then attention is paid in particular to Eringen’s model [101] for generating the multiplicative damage factor, related to quasi-static analysis. In the first step, the XFEM is used, then the stress in front of the crack is recalculated according to the nonlocal approach in the entire body according to the exponential law of violation. The following Figure 14 and Figure 15 present some comparative results.

For most numerical tests, a slightly modified method of the extended finite element method was used; this method uses new degrees of freedom for crack propagation within the standard element (extrinsic XFEM). The test material, in our case, a cement paste reinforced with metal fibres, had the following material characteristics: Young’s modulus E = 3.2 GPa, Poisson’s ratio μ = 0.3 and tensile strength 12 MPa. Using a nonlocal approach to calculate the stress value in front of the crack will significantly change the crack propagation rate in the model case. Accelerating the damage with a damage parameter higher than 0.2 creates the preconditions for multiple small cracks around the stress concentrator.

In some complicated situations, ignoring crack tip effects, the stability of the XFEM discretisations is trivial for open cracks but remains a challenge if we constrain the crack to be closed (i.e., the bi-material problem [95]). The demand for lightweight and high-strength materials in the aerospace, automotive and marine industries has necessitated the use of fibre-reinforced polymeric composites in place of metal alloys [116]. Related to the mentioned issue is the issue of complex loading, or in the case of the aviation industry, also modelling the response to cyclic loading, [117]. Recently, there have even been articles focusing on radiation damage and modelling the formation of cracks inside solar cells, e.g., in [118]. Indeed, almost unexpected is the use of cohesion elements to estimate the response of human tissue when inserting deep needles into soft tissues in [119], or for the development of bone-inspired composites in [120].

## 4. Conclusions

The conclusions can be characterised by the following:

- The procedure for implementing the cohesion element into the FEM system was indicated.
- With the advent of fibre composites in technical practice, it is essential to be able to predict or model the behaviour of these materials. Not only must numerical methods solve new or modified procedures, including the existence of solutions, but the modelling result must also clearly approach reality. The problem is that many of the input data are estimated, which increases the risk of a possible wrong prediction.
- An example of numerical problems introduced is the form of the traction–separation law in cohesion models.
- Talking about the fineness of the FEM network can be counterproductive; it is necessary to start with the size of the RVE (representative volume element).
- Small modifications of XFEM with a focus on the applicability of these procedures were also tested on practical examples.
- For the modelling of microstructural behaviour using XFEM, it is often necessary to use, or rather to introduce, a real traction–separation law.
- Careful determination of the traction–separation law representing all phases of fibre reinforced composite behaviour enables a more accurate prediction of crack propagation predominantly in the initial phase of failure.
- In the case of cement composites, it is reasonable to use models that in a certain way average the stress field in front of the crack.
- The combination of the traction–separation law and XFEM is a promising approach for crack propagation modelling and is a strong motivation for further research.
- Three groups of materials were tested: ductile steel, ceramic composites and cementitious composites reinforced with metal fibres. From a microstructural point of view, the materials have different RVE sizes but the procedures and modelling of crack initiation and propagation using XFEM and using the cohesive approach are very similar.
- The outlined path is probably promising for the future higher industrial deployment of these fibre composites. For example, in the field of building composites, the production of building components with directed fibres in the given component is expected, which will enable production with clear, predictable properties.
- The theory of sharp stress concentrators in front of the crack tip for the named types of materials is abandoned, and so-called smeared models with averaged stress are promising and could be a subject of future research, as presented in this paper.

Some more comments: Remarkable progress in engineering in the last decades is connected with the use of advanced materials, whose design for implementation in engineering structures cannot rely on long-time experience, justifying the simplified computational formulae. A proper physical formulation covering elastic, viscous and plastic deformation including damage for a sufficiently wide class of materials— including those three discussed in Section 2, taking all available information about material structure into account, together with relevant additional physical, chemical, etc. processes— is still a research challenge for the near future. It should be accompanied by the mathematical analysis of existence, uniqueness, smoothness and other important properties of solutions of related evolutionary problems for partial differential equations, as well as by the development of robust and effective computational algorithms, reflecting the progress in computer hardware and software, as in parallel and distributed systems, artificial intelligence, etc.

## Data Availability

The data presented in this study are available on request from the corresponding author.
